# An Improved Deep Learning Algorithm for Breast Cancer Survival Prediction Based on Multi-Omics Data

**DOI:** 10.12688/f1000research.166682.2

**Published:** 2025-08-29

**Authors:** Nurul Athirah Nasarudin, Fatma Al-Jasmi, Nor Hidayati Abdul Aziz, Nor Azlina Ab Aziz, Wasif Khan, Yusuf Hendrawan, Dimas Firmanda Al Riza, Ayisha Manzoor, Bassam R. Ali, Mohd Saberi Mohamad

**Affiliations:** 1 Department of Genetics and Genomics, College of Medicine and Health Sciences, United Arab Emirates University, Al Ain, Abu Dhabi, 17666, United Arab Emirates; 2 Centre for Advanced Analytics, CoE for Artificial Intelligence, Multimedia University, Malacca, Malacca, 75450, Malaysia; 3 Faculty of Engineering & Technology, Multimedia University, Malacca, Malacca, 75450, Malaysia; 4 J. Crayton Pruitt Family Department of Biomedical Engineering, Herbert Wertheim College of Engineering, University of Florida, Gainesville, Florida, 32 611, USA; 5 Department of Biosystems Engineering, Faculty of Agricultural Technology, Universitas Brawijaya, Malang, East Java, 65145, Indonesia

**Keywords:** Artificial Intelligence, BiLSTM, Breast Cancer, CNN, Deep Learning, Multi-omics

## Abstract

**Background:**

Breast cancer is a leading cause of mortality among women worldwide. Accurate survival prediction can improve clinical decision-making and support personalized treatment planning. This study aims to develop an interpretable and effective deep learning model for breast cancer survival prediction using multi-omics data.

**Methods:**

This study proposes a novel deep learning model combining Bi-directional Long Short-Term Memory (BiLSTM) and Convolutional Neural Network (CNN) architectures, integrated with Minimum Redundancy Maximum Relevance (MRMR) feature selection. The model was evaluated on two large datasets: METABRIC (n=1980) and TCGA-BRCA (n=1080), using clinical, copy number alteration (CNA), and gene expression data. Performance was assessed through metrics such as AUC-ROC and accuracy.

**Results:**

The proposed model demonstrated superior performance compared to existing algorithms, achieving high AUC-ROC and accuracy values across all data modalities. The integration of BiLSTM and CNN architectures allowed the model to capture temporal and spatial patterns, improving prediction robustness. Notably, the model achieved an accuracy of 98% on the METABRIC dataset and 96% on the TCGA dataset.

**Conclusions:**

The combination of BiLSTM, CNN, and MRMR offers an interpretable and accurate framework for breast cancer survival prediction using multi-omics data. This approach provides actionable insights for clinicians and highlights its potential for broader applications in oncology.

## Introduction

Breast cancer is a heterogeneous disease and one of the leading causes of cancer-related death among women all over the world. ^1^ According to GLOBOCAN 2018, 11.6% of 9.6 million cancer cases were breast cancer, making it the most diagnosed. ^1^ This pattern is also found in developed countries, with an incidence rate of 54.5 per 100,000 women, especially in areas with a high Human Development Index (HDI). ^2^ Typically, breast cancer occurs in middle-aged and older women, but in recent times, cases among younger women under 40 have also been reported. Breast cancer in younger women often presents with more advanced stages and worse outcomes, contributing to higher mortality rates. ^3^ Early diagnosis and treatment improve survival rates significantly, emphasizing the need for accurate prognostic models. ^4^ The advent of high-throughput omics technologies allows researchers to explore complex diseases by measuring thousands of biological molecules simultaneously. ^5^ Combining multi-omics data with clinical features provides valuable insights into cancer progression and treatment responses, paving the way for predictive modeling of survival outcomes. Publicly available datasets like METABRIC and TCGA-BRCA have become essential resources for breast cancer research, enabling the development of survival prediction models. ^6^

Despite advances in predictive modeling, challenges remain. Traditional machine learning models often function as “black-box” models, limiting their clinical utility due to the lack of interpretability. Clinical decision-making requires models that provide transparent, actionable insights alongside accurate predictions. Additionally, the high-dimensionality of multi-omics data increases the risk of overfitting, making it difficult for models to generalize effectively across different patient cohorts. There is a critical need for models that balance interpretability, predictive accuracy, and computational efficiency to address these challenges.

Our study aims to bridge this gap by developing a novel deep learning model that integrates Bi-directional Long Short-Term Memory (BiLSTM) and Convolutional Neural Networks (CNN) for feature extraction, along with MRMR (Minimum Redundancy Maximum Relevance) feature selection to reduce dimensionality and enhance interpretability. This approach offers improved prediction accuracy and actionable insights, making the model more suitable for clinical adoption. In contrast to previous studies that rely primarily on clinical data, our model leverages multi-omics data (e.g., gene expression, DNA methylation, miRNA, and copy number alterations) to enhance predictive performance.

Several studies have explored the potential of machine learning models for survival prediction. For example, Zhao et al. ^7^ combined gene expression data with clinical and pathological factors, achieving AUC values of 0.72 and 0.67 using artificial neural networks (ANN) and support vector machines (SVM). Goli et al. ^8^ employed support vector regression for survival prediction, with promising results for imbalanced datasets. Gevaert et al. ^9^ used Bayesian networks to combine microarray gene expression data with clinical information, achieving a maximum AUC of 0.845. Sun et al. ^10^ improved predictive performance by integrating genomic and imaging data, achieving an AUC of 0.828 ± 0.034. Ma and Zhang ^11^ applied factorization autoencoders to multi-omics data, achieving AUCs of 0.74 and 0.825 for bladder cancer and brain glioma, respectively. Experienced medical professionals face challenges in treating invasive breast cancer because it is difficult to synthesize and analyze large amounts of data from multiple sources. ^12^ The increasing availability of omics data offers new opportunities for creating predictive algorithms but introduces challenges related to data integration, heterogeneity, and high dimensionality. ^5^

This research presents a novel deep learning algorithm combining BiLSTM and CNN architectures for survival prediction, validated using METABRIC and TCGA datasets. In addition to its predictive accuracy, the model offers interpretability through feature importance analysis, enhancing its relevance for clinical decision-making. By setting a five-year survival threshold, the model classifies patients as short-term or long-term survivors, supporting physicians in tailoring treatment plans and minimizing unnecessary interventions.

Through the decision-level integration of multi-omics data, this study addresses challenges related to high-dimensionality, overfitting, and model interpretability. The results demonstrate significant improvements over existing algorithms, offering a pathway toward more transparent, clinically applicable predictive models. The findings contribute to the growing field of personalized oncology, paving the way for future research and the development of prognostic tools for breast cancer.

### Significance of the study

Survival prediction in breast cancer remains a complex task due to the intrinsic high dimensionality, noise, and heterogeneity of multi-omics datasets, which pose significant challenges for conventional predictive models. Existing machine learning methods often fail to fully leverage the complementary information embedded across different omics layers and struggle to generate clinically interpretable outputs. In this study, we propose a hybrid BiLSTM+CNN deep learning architecture that effectively captures both temporal dependencies and hierarchical feature representations within integrated multi-omics data. The model demonstrates superior predictive performance on benchmark METABRIC and TCGA datasets, while incorporating interpretability mechanisms to enhance clinical relevance. By addressing both the data integration and interpretability bottlenecks, this work provides a robust and scalable framework for precision oncology applications, offering improved survival prediction capabilities that can directly inform personalized treatment strategies.

## Methods

### A. Datasets

This study uses the METABRIC breast cancer dataset, consisting of 1980 patient records, available through the cBioPortal database ( https://www.cbioportal.org/study/summary?id=brca_metabric). ^13^ The cBioPortal offers a web-based platform for exploring and visualizing multidimensional cancer genomics data, converting complex molecular profiling from cancer tissues and cell lines into readily understandable genetic, epigenetic, gene expression, and proteomic information. The dataset contains information from three data modalities: clinical profile, gene expression profile, and copy-number alteration (CNA) profile. Patients were grouped based on their survival outcomes into two categories: long-term survivors (≥5 years) with 1489 samples (labeled as ‘0’), and short-term survivors (<5 years) with 491 samples (labeled as ‘1’). The median age at diagnosis for patients is 61 years, with an average survival duration of 125.1 months. Table 1 summarizes the key characteristics of the METABRIC dataset.

**Table 1.** Summary of the METABRIC dataset.

Details	Records
Disease	Breast cancer
Number of patients	1980
Survival time (years)	5
Survival > (5 years)	1489
Survival < (5 years)	491
Number of modalities	3
Modalities	Clinical, Gene Expression, and CNA profile

The clinical features available in the METABRIC dataset include age at diagnosis, tumor size, estrogen receptor status, HER2 status, and stage at diagnosis. During pre-processing, two of the original 27 clinical features were removed due to missing data and redundancy, reducing the number of clinical features to 25. This feature reduction ensures that only the most relevant variables are retained, enhancing the predictive capacity of the proposed model. To provide deeper insights, a univariate t-test analysis was conducted on key clinical features to assess how they differ between short-term and long-term survivors. The results of the descriptive statistics and t-tests are presented in Table 2 below. Although the METABRIC dataset contained 25 clinical features after preprocessing, the t-test analysis highlighted a subset of widely recognized prognostic indicators, including age, tumor size, estrogen receptor status, HER2 status, and stage at diagnosis. These variables were chosen because they are consistently associated with breast cancer survival outcomes and provide clinically interpretable insights, as also emphasized in prior studies. ^14^ The remaining clinical variables were retained for model training but are not individually reported here, as they exhibited limited statistical differentiation between survivor groups.

**Table 2.** Descriptive statistics and univariate t-test results for clinical data in METABRIC dataset.

Clinical feature	Short-term survivors (Mean ± SD)	Long-term survivors (Mean ± SD)	t-value	p-value
Age at Diagnosis (years)	55.3 ± 10.1	63.2 ± 9.8	2.23	0.03
Tumor Size (cm)	4.2 ± 1.5	2.8 ± 1.3	2.71	0.01
Estrogen Receptor (%)	60 ± 12	72 ± 10	2.08	0.04
HER2 Status (%)	50 ± 15	48 ± 12	0.98	0.12
Stage at Diagnosis	3.1 ± 0.8	2.5 ± 0.6	2.58	0.02

As shown in Table 2, several clinical features differ significantly between the two survivor groups. Long-term survivors tended to be older at the time of diagnosis, with a mean age of 63.2 years, compared to 55.3 years for short-term survivors (p = 0.03). In addition, tumor sizes were notably smaller among long-term survivors (2.8 cm) compared to those in the short-term group (4.2 cm), with a p-value of 0.01. Similarly, the stage at diagnosis was lower for long-term survivors, indicating early detection and more favorable prognoses (p = 0.02). However, HER2 status showed no statistically significant difference (p = 0.12), suggesting it may not directly influence survival outcomes. These findings emphasize the role of early detection, tumor size, and diagnosis stage in predicting long-term survival, which are essential factors for clinical decision-making.

The heterogeneity in the METABRIC dataset reflects the merging of data from multiple hospitals, leading to variability in clinical practices, treatment protocols, and laboratory standards. In addition, patients often received concomitant medications alongside primary treatments, such as supplements or medications to manage side effects. These factors underscore the complexity of predicting survival outcomes in breast cancer patients. To further validate the model’s generalizability, the TCGA-BRCA dataset was employed. This dataset, available through the GDC portal ( https://portal.gdc.cancer.gov/projects/TCGA-BRCA ), contains 1080 patient records with the same three data modalities as METABRIC—clinical profile, gene expression profile, and copy-number alteration (CNA) profile. ^15^ Table 3 below summarizes the details of the TCGA-BRCA dataset.

**Table 3.** Summary of the TCGA-BRCA dataset.

Details	Records
Disease	Breast cancer
Number of patients	1080
Survival time (years)	5
Survival > (5 years)	250
Survival < (5 years)	830
Number of modalities	3
Modalities	Clinical, Gene Expr, and CNA profile

### B. Data augmentation

Deep learning (DL) models have demonstrated remarkable achievements in tasks involving histological images, topographies, and clinical data. However, their performance with gene expression data remains constrained due to the complex nature and high dimensionality of such datasets, which often require thousands of instances to achieve reliable outcomes. To mitigate these challenges, Data Augmentation (DA) techniques can be adapted for transcriptomic data, although their application is less common than in imaging tasks. In this study, we employed noise injection as augmentation strategies. Noise injection has been shown to be particularly effective for omics data, as it simulates the measurement variability inherent in RNA-seq and microarray platforms while generating synthetic yet biologically plausible training instances. Prior studies such as Islam et al. demonstrated that injecting noise into gene expression features improved classification robustness across cancer datasets. ^16^ Additionally, gene expression data introduces challenges due to its variability and susceptibility to the curse of dimensionality—where datasets contain more features than available samples. As a result, DA techniques play a pivotal role in increasing the size of training datasets by generating synthetic data samples, thereby improving the generalization capacity of models.

In this study, random rotation and noise injection techniques were applied to gene expression data as DA methods. The noise injection technique involved randomly selecting training samples and altering up to 25% of their features. The noise was generated from a normal distribution with a standard deviation of 0.2 and was added to the original feature values. To ensure data validity, the modified values were clamped within the range of [0, 1]. The selected standard deviation value (0.2) ensured that the augmented samples remained close to the original data instances. Random rotation, on the other hand, was adapted to the feature space rather than physical geometry. Each gene expression profile was treated as a high-dimensional vector, and random orthogonal transformations were applied to rotate these vectors while preserving their variance and overall structure. This produced synthetic samples that retained the statistical properties of the dataset but in alternative orientations of the feature space. Such transformations are conceptually related to PCA-based rotations and latent-space perturbations used in omics DA studies. ^16^^,^^17^ From a biological perspective, these augmentations capture natural variability in gene–gene correlation structures across patients or cohorts, helping the model learn robust interaction patterns instead of relying on fixed dependencies. A similar idea was demonstrated by previous study, where latent-space mixing in single-cell RNA-seq generated biologically plausible samples that enhanced generalization. ^17^ Recent research on DL models for genomic datasets highlights the potential benefits of DA techniques, although the application of DA to genomic data remains relatively unexplored. ^18, 19^ The integration of DA techniques in this study addresses imbalances in gene expression data and enhances the predictive capabilities of the model by preventing overfitting to limited sample sizes.

### C. Pre-processing data

This study utilized three key data modalities: clinical profile, gene expression profile, and copy-number alteration (CNA) profile. Each of these datasets underwent a thorough pre-processing pipeline to ensure data quality and consistency for analysis. The pre-processing steps involved handling missing values, normalization, feature discretization, and feature selection, all of which were necessary to prepare the data for the deep learning algorithm.

To address missing values in the gene expression and CNA datasets, the weighted nearest neighbor (KNN) algorithm was employed. ^20^ This algorithm imputed unknown values by evaluating the closest known neighbors, ensuring consistency with the original data structure. After the imputation, the datasets were normalized to maintain a consistent scale for all features. ^9^ Following normalization, the CNA data was discretized into five categories: −2, −1, 0, 1, and 2, representing varying levels of copy number variation. Similarly, the gene expression values were categorized into three classes: -1 (under-expressed genes), 0 (baseline genes), and 1 (over-expressed genes). These discretization steps ensured that the features were more interpretable and ready for machine learning processing.

Given the high-dimensional nature of both the CNA and gene expression datasets, feature selection was essential to reduce the dimensionality and improve the model’s generalizability. The MRMR (Maximum Relevance—Minimum Redundancy) algorithm was selected for this task due to its ability to reduce redundancy while retaining the most relevant features. Alternative methods, such as LASSO regression and Principal Component Analysis (PCA), were considered; however, MRMR was chosen because it provided better interpretability and reduced the risk of overfitting in our experiments. Furthermore, hyperparameter tuning was applied to the MRMR process to ensure the most informative features were selected for the final model. Among the three modalities used in this study, copy number alteration (CNA) and gene expression data posed significant dimensionality challenges, with each CNA sample containing over 26,000 features and gene expression profiles exceeding 24,000 features. When the number of features exceeds the number of observations, models tend to overfit easily, a problem commonly known as the curse of dimensionality. ^21^ The feature selection process was performed in a gradational strategy, where subsets of features were generated in increments of 100 and evaluated using AUC as the selection criterion. The algorithm was executed across feature set sizes ranging from 100 to 500, and the subset yielding the highest validation performance was chosen. This tuning process identified 400 features for gene expression and 200 features for CNA as optimal, balancing predictive accuracy with model generalizability. The clinical dataset, which contained 27 initial variables, was reduced to 25 key features after preprocessing. These included well-known prognostic indicators such as hormone receptor status, tumor size, menopausal state, lymph node positivity, histological grade, treatment type, and surgical information, all of which have established relevance to breast cancer survival outcomes.

From the CNA profile, the feature count was reduced from 26,298 to 200, and from the gene expression profile, it was narrowed down from 24,368 to 400. For the clinical dataset, the original 27 features were reduced to 25 after removing two features with missing data. The resulting pre-processed dataset, summarized in Table 4 below, served as the input for the proposed deep learning algorithm, enabling more accurate survival predictions.

**Table 4.** Pre-processed data.

Data modality	Total features	Selected features
Clinical	27	25
CNA profile	26298	200
Gene expression	24368	400

### D. Convolutional Neural Network (CNN)

Convolutional Neural Network (CNN) is a type of feed-forward neural network widely used for tasks involving image processing, natural language processing (NLP), and time series data prediction. ^22^ One of the key advantages of CNN is its local perception mechanism and weight sharing across different layers. This design significantly reduces the number of parameters, thereby improving the model’s efficiency in training and generalization. A typical CNN model is composed of three essential components: the convolution layer, pooling layer, and fully connected layer. The convolution layers extract relevant features from the input data, though the extracted features may have a high dimensionality. To address this, a pooling layer is applied after each convolution layer, which reduces the feature dimensions and computational cost while retaining the most important information.

While CNN has demonstrated exceptional performance in many domains, it has limited capacity to process large-scale, multi-modal data such as genomic and clinical datasets. Recently, researchers have focused on multi-source data integration to enhance the predictive capabilities of deep learning models. These advanced deep learning algorithms that combine multiple data modalities exhibit superior performance over models that rely solely on a single data source. The integration of CNN into such frameworks makes it a promising tool for tasks like cancer survival prediction by efficiently capturing feature patterns across different data types.

### E. Bi-Directional LSTM (BiLSTM)

The traditional Long Short-Term Memory (LSTM) network, though effective in modeling sequential data, processes information in only one direction—either forward or backward in the sequence. ^23^ This limitation can hinder the model’s ability to fully capture the temporal dependencies inherent in sequential datasets. To overcome this, the Bi-directional Long Short-Term Memory (BiLSTM) network was developed, enabling the processing of information in both directions—forward and backward. The core idea of BiLSTM is to analyze sequences both front-to-back and back-to-front. In this model, one LSTM layer processes the sequence from the start to the end, while another layer processes it from the end to the start. This dual-directional processing allows the network to retain information from both past and future contexts, making it particularly useful for analyzing time series data and sequential inputs from multi-omics datasets.

In this study, the input data from multi-omics sources is first processed by two BiLSTM layers in the initial module. The extracted features are then passed to the CNN layers in the subsequent module for further feature extraction and dimensionality reduction. The combined BiLSTM and CNN architecture ensures that both the temporal dependencies and spatial patterns in the data are captured. In the final stage, the fully connected layers generate predictions about patient survival, classifying breast cancer patients as either short-term or long-term survivors.

### F. An improved deep learning algorithm: BiLSTM and CNN algorithm for prediction using multi-omics data

**Figure 1.**
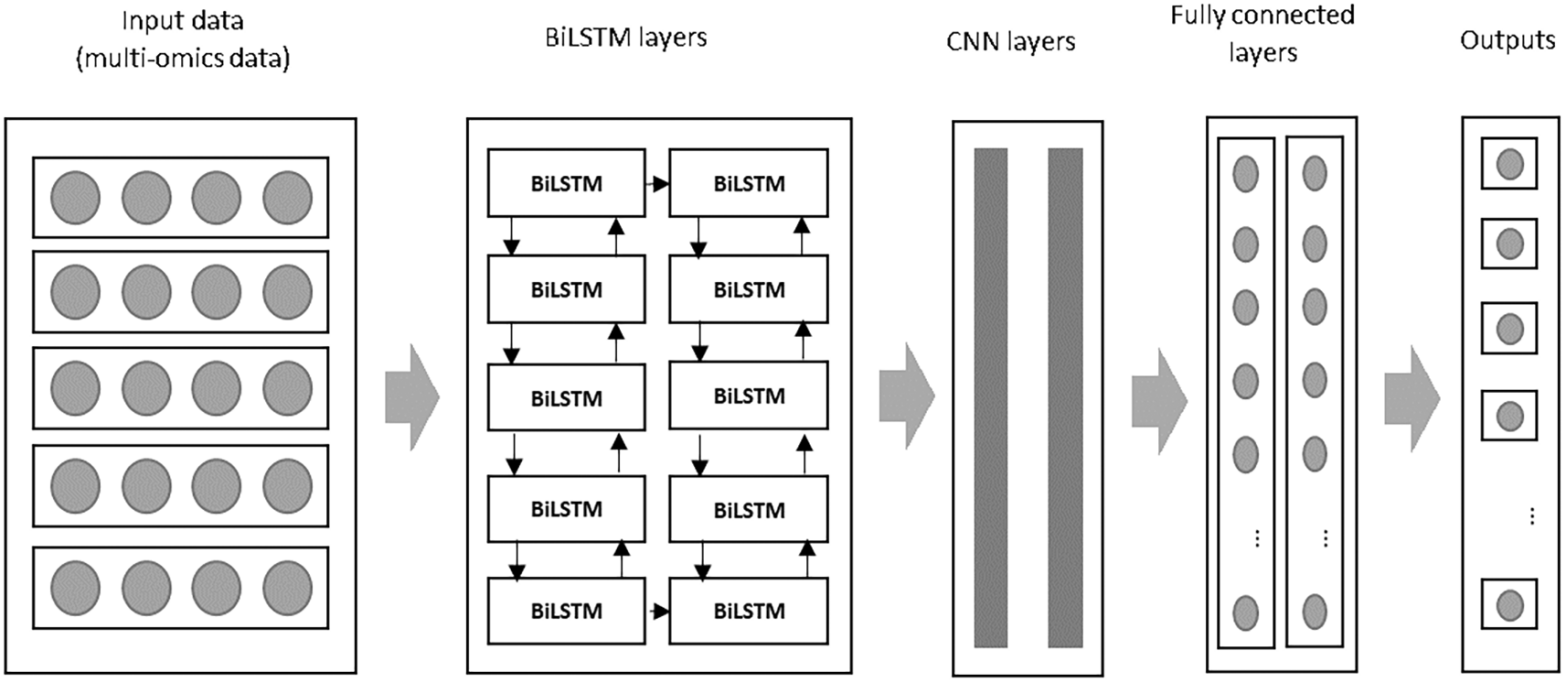
An overview of the proposed improved deep learning algorithm namely BiLSTM+CNN.

This study presents an improved deep learning algorithm by integrating a Bi-directional Long Short-Term Memory (BiLSTM) network with a Convolutional Neural Network (CNN) to predict breast cancer survival and extract meaningful features from multi-omics data. The BiLSTM addresses the limitations of traditional LSTM networks, while CNN complements it by capturing the spatial patterns in the data. The proposed combination offers superior performance by leveraging the strengths of both models: BiLSTM for temporal sequence learning and CNN for feature extraction. An overview of the proposed model is illustrated in Figure 1 below:

During the initial phase, both BiLSTM and CNN layers are configured with specific filters to extract key features. These features are processed through convolution and dense layers, generating a feature map that feeds into subsequent stages of the model. As described in, ^14^ the Glorot normal initializer is used to initialize the filter values, ensuring that the parameters follow a normal distribution with a mean of zero. A fixed seed value of 0.1 is used to maintain consistency in model training, preventing variation in results between different runs.

Hyperparameter tuning was conducted using a grid search approach. The key parameters tuned included the number of layers, filter sizes, learning rate, and regularization strength. For each parameter combination, model performance was evaluated on the validation set using AUC-ROC as the primary metric. The final configuration (outlined in Table 5) was selected based on the highest AUC and accuracy scores observed during cross-validation. This tuning process ensured optimal performance while preventing overfitting. The parameters and architecture of the BiLSTM+CNN algorithm is detailed in Table 5 below:

**Table 5.** Architecture and parameter related details of BiLSTM+CNN algorithm.

Component	Parameter	Details
BiLSTM Layer	LSTM Layers	2
	Number of Hidden Units	32
Convolutional Layer	Convolutional Layers	2
	Filter Size	15 × 15
	Number of Filters	25
	Stride Size	2
	Padding	Same
	Activation Function	ReLU
Fully Connected Layer	Number of Hidden Layers	2
	Hidden Units in Each Layer	150, 100
	Activation Function	TANH
Output Layer	Activation Function	Sigmoid
Training Configuration	Number of Training Epochs	20
Loss Function	Loss Function Used	Binary Cross-Entropy + L2 Regularization

Below is an overview of the BiLSTM+CNN algorithm:

Algorithm 1. **BiLSTM+CNN.**

**Input** Dataset (Clinical, CNA, Gene exp), number of epochs N, number of folds K

**Output** Extraction features

1. Initialize the BiLSTM+CNN algorithm with the required parameters.
2. Perform train-test split: TrainData, TestData.
3. Partition TrainData into K subsets F _1_, …, F _K_.
4. **For k = **1 to **K**: • Data_train = dataset −Fk • Data_valid = Fk
5. For epoch e = 1 to N: • Train BiLSTM+CNN using Data_train. • Validate the model with Data_valid.
6. Test the model using TestData.
7. End Procedure.

The pre-processed data serves as input for the BiLSTM layers, whose outputs are subsequently passed to the CNN layers for further feature transformation. Within the convolutional layers, a stride size of 2 is employed, meaning the filter shifts by 2 units across the input matrix during the convolution operation. Padding is applied to ensure that feature sizes remain consistent. A flattened layer follows, converting the multi-dimensional output into a format suitable for the dense layer, which comprises 150 hidden units. To mitigate overfitting, L2 regularization is incorporated within the CNN model. ^24^ The activation functions used include ReLU for the convolutional layer and sigmoid for the dense layer. The Adam optimizer ^25^ was employed for optimization, with binary cross-entropy serving as the loss function for the binary classification task of predicting patient survival.

The proposed BiLSTM+CNN algorithm is divided into three phases:

• Phase 1: The algorithm is trained using clinical data, CNA data, and gene expression data.
• Phase 2: A stacked feature set is created from the extracted features of the BiLSTM+CNN model.
• Phase 3: The stacked feature set is passed through a Random Forest (RF) algorithm for final classification. The architecture of the BiLSTM+CNN

**Figure 2.**
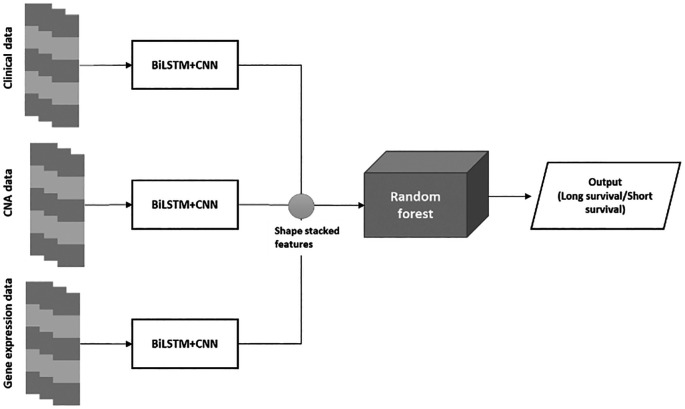
BiLSTM+CNN stacked RF architecture.

Stacked RF model is depicted in Figure 2 below:

The final classification employed a Random Forest (RF) model with n_estimators = 200, max_depth = None, random_state = 0, and class_weight = ‘balanced’. These parameters were selected through grid search, ensuring the best AUC and accuracy scores during cross-validation. This configuration offered the optimal balance between precision and recall, especially for the imbalanced classes in our dataset. Using class_weight = ‘balanced’ mitigated the risk of overlooking minority classes, while setting max_depth = None enabled the model to capture complex feature interactions without overfitting.

### G. Performance evaluation and metrics

The performance of the proposed BiLSTM+CNN algorithm was evaluated using several metrics, including Sensitivity, Specificity, Precision, and Accuracy and the Area Under the Curve (AUC) of the Receiver Operating Characteristic (ROC) curve. The metrics are defined as follows:

$$\text{Sensitivity}=\frac{\mathrm{Tp}}{\mathrm{Tp}+\mathrm{Fn}}$$ (1)

$$\text{Specificity}=\frac{\mathrm{Tn}}{\mathrm{Tn}+\mathrm{Fn}}$$ (2)

$$\text{Precision}=\frac{\mathrm{Tp}}{\mathrm{Tp}+\mathrm{Fp}}$$ (3)

$$\text{Accuracy}=\frac{\mathrm{Tp}+\mathrm{Tn}}{\mathrm{Tp}+\mathrm{Tn}+\mathrm{Fp}+\mathrm{Fn}}$$ (4)

Here, TP (true positive), TN (true negative), FP (false positive), and FN (false negative) denote the classification outcomes. Additionally, the AUC-ROC curve assesses the model’s ability to distinguish between classes across various thresholds, providing a comprehensive view of performance beyond a single point metric.

### H. Cross validation

The ten-fold cross-validation approach was adopted for model evaluation, following recommendations from prior studies. ^14^ In this method, the dataset is randomly divided into ten equal subsets. For each fold, nine subsets are used for training, while one subset is held out for testing. This process ensures that every data point is used for both training and testing, thereby providing a more reliable performance estimate.

Within each merged training set, 80% of the data is allocated for training the model, while the remaining 20% is reserved for validation to fine-tune hyperparameters and prevent overfitting. The Keras and TensorFlow libraries were employed for model implementation, ensuring computational efficiency and ease of experimentation.

## Results

### A. Performance of The Improved Deep Learning Algorithm (BiLSTM+CNN)

**Figure 3.**
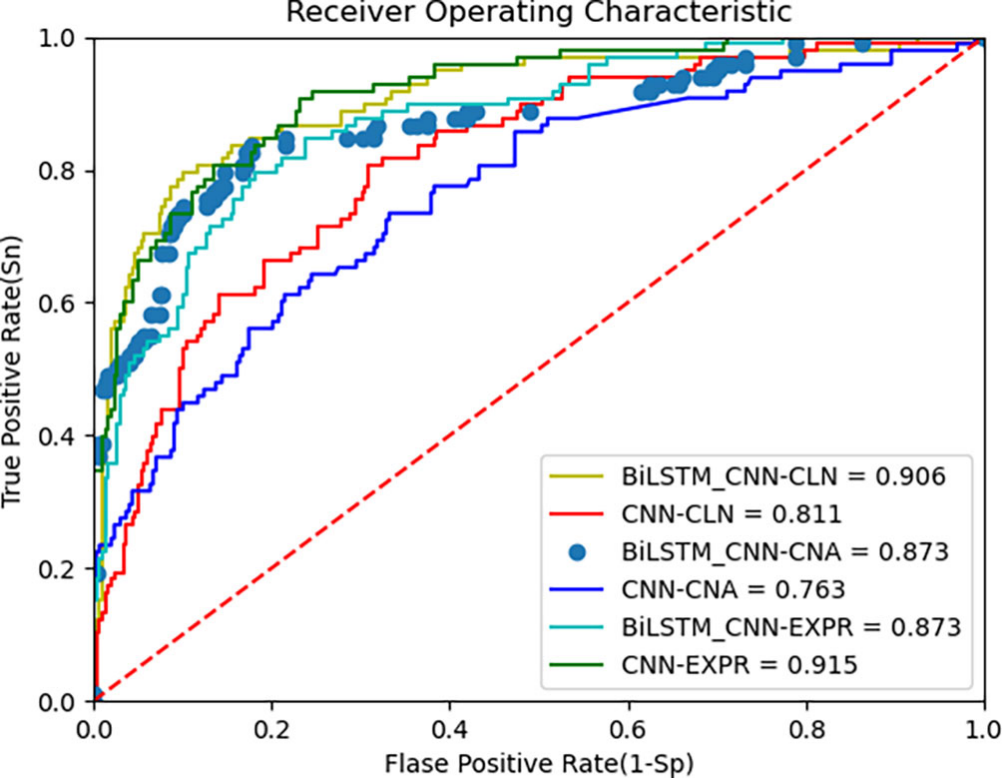
ROC curve of improved deep learning algorithm (BiLSTM+CNN) and CNN as feature extractors on METABRIC data.

The proposed deep learning algorithm leverages BiLSTM and CNN for feature extraction from multi-omics data. The AUC metric from the ROC curve, along with accuracy, is used to evaluate the model’s performance. Figure 3 below shows the ROC curves of the BiLSTM+CNN compared to CNN for the METABRIC dataset. The AUC values are 0.90, 0.87, and 0.87 for clinical data, CNA, and gene expression data, respectively. To provide a more comprehensive view of the model’s performance, we report the 95% confidence intervals (CI) for each modality. These CIs offer an estimate of variability across different trials, ensuring more reliable interpretation of results:

• Clinical data: Accuracy = 0.90, 95% CI [0.9027, 0.8973]
• CNA data: Accuracy = 0.867, 95% CI [0.872, 0.868]
• Gene expression data: Accuracy = 0.876, 95% CI [0.8812, 0.8788]

These results indicate the model’s robustness across different data modalities, though performance on gene expression data is slightly lower, reflecting the challenges posed by high-dimensional data. The third plot in the above Figure 3 is presented with data points rather than a continuous line to highlight specific thresholds along the ROC curve. This visualization helps illustrate how key decision points, such as cutoff thresholds, impact the true positive (TPR) and false positive rates (FPR). Disconnected lines may occur due to discrete prediction values or gaps in the input data, especially when thresholds do not span the full range of possible values. While this approach enhances interpretability, future iterations could explore smoothing techniques to provide a continuous curve.

### B. Addressing model limitations

Our model’s false positive rate was higher than expected, which could have clinical implications. To address potential overfitting and variance due to the small dataset size, we applied ten-fold cross-validation. The 1980-patient dataset was split into ten subsets, with nine subsets for training and one for testing. Each training set was further divided into 80% for training and 20% for validation.

We combined extracted features from BiLSTM and CNN into a stacked feature set, which was then classified using a Random Forest (RF) algorithm. As previous studies show, RF performs better with stacked features compared to other classifiers. ^10^ Performance metrics, including sensitivity, specificity, and precision, were calculated to assess the model’s effectiveness.

### C. ROC curve and comparison with other algorithms

Figure 3 above presents the ROC curve comparing BiLSTM+CNN and CNN feature extractors on METABRIC data. The results demonstrate superior AUC values for BiLSTM+CNN across different modalities. Table 6 below summarizes the comparison of AUC and accuracy with existing algorithms:

**Table 6.** Comparison of improved deep learning algorithm (BILSTM+CNN).

Algorithm		AUC	ACC
BiLSTM+CNN Stacked RF	BiLSTM+CNN-clinical	**0.91**	**0.88**
	BiLSTM+CNN-cna	**0.87**	**0.85**
	BiLSTM+CNN-gene expression	0.87	0.82
Heterogenous Stacked RF [28]	CNN-clinical	0.85	0.81
	DNN-cna	0.72	0.80
	CNN-gene expression	0.90	0.80
Stacked RF [28]	CNN-clinical	0.83	0.80
	CNN-cna	0.75	0.74
	CNN-gene expression	0.92	0.80
MDNNMD [28]	DNN-clinical	0.81	0.79
	DNN-cna	0.61	0.76
	DNN-gene expression	0.76	0.74
SiGaAtCNN Stacked RF [8]	SiGaAtCNN-clinical	0.86	0.81
	SiGaAtCNN-cna	0.83	0.84
	SiGaAtCNN-gene expression	**0.95**	**0.89**

The bold values in Table 6 highlight the best performing results for each data modality (clinical, CN and gene expression) across all compared algorithms. The results clearly demonstrate that the proposed BiLSTM+CNN algorithm performs better than previous algorithms across multiple data modalities. However, in the gene expression modality indicate that the SiGaAtCNN Stacked RF model achieved superior performance, outperforming the proposed BiLSTM+CNN model in that specific category. The comparison included models such as MDNNMD, SiGaAtCNN, and Heterogeneous Stacked RF. As shown in Table 7 below, our algorithm outperforms others in terms of accuracy, precision, sensitivity, and Matthews correlation coefficient (MCC).

**Table 7.** Comparison of classification performance of Improved Deep Learning Algorithm (BiLSTM+CNN) with previous works on METABRIC data.

Algorithm	Acc	Pre	Sn	Mcc
BiLSTM+CNN Stacked RF	0.98	0.95	1.0	0.81
Heterogenous Stacked RF [28]	0.97	0.98	0.97	-
Stacked RF [8]	0.90	0.84	0.75	0.73
MDNNMD [28]	0.83	0.75	0.45	0.47
SiGaAtCNN Stacked RF [8]	0.91	0.84	0.80	0.77

### D. Validation on TCGA dataset

To further validate the performance, we used the TCGA-BRCA dataset. ^15^ This dataset contains 250 long-term survivors and 830 short-term survivors, with data modalities matching those in the METABRIC dataset. Pre-processing was conducted using the same steps outlined in Sections B and C.

**Figure 4.**
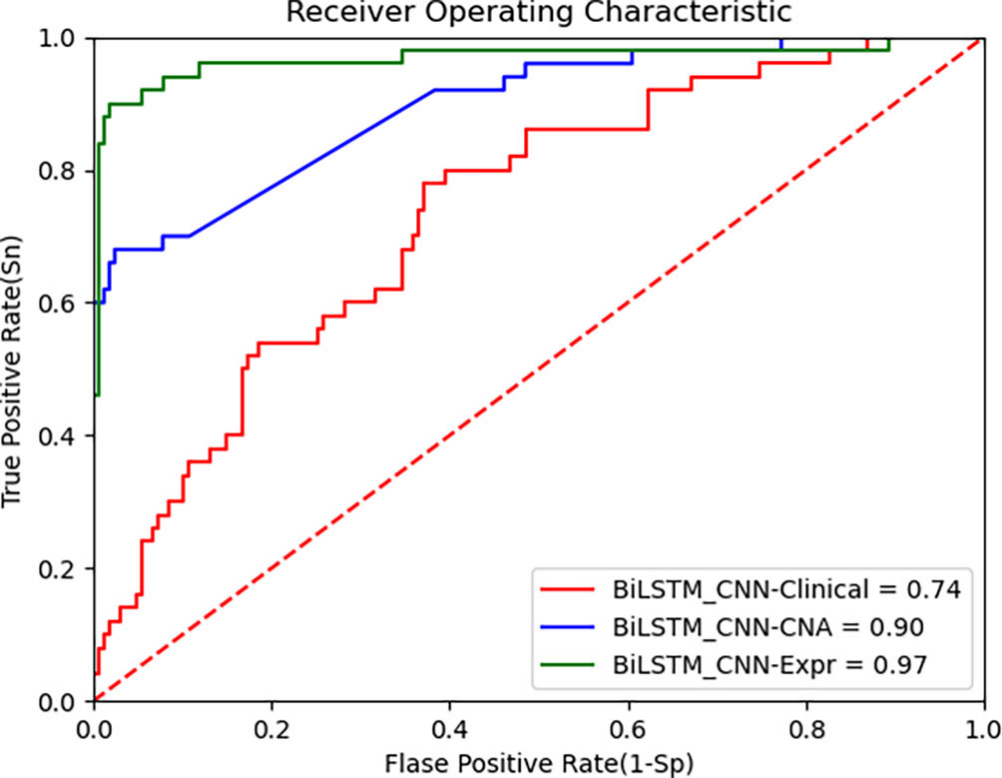
ROC curve of improved deep learning algorithm (BiLSTM+CNN) and CNN as feature extractors on TCGA data.

Figure 4 presents the ROC curve for the TCGA dataset, demonstrating that the BiLSTM+CNN Stacked RF algorithm maintains high performance across datasets. Below are the performance metrics along with the 95% confidence intervals (CI):

• Clinical data: Accuracy = 0.739, 95% CI [0.741, 0.737]
• CNA data: Accuracy = 0.903, 95% CI [0.906, 0.900]
• Gene expression data: Accuracy = 0.964, 95% CI [0.965, 0.962]

These results show that the model generalizes well to the TCGA dataset, especially on gene expression data, where it achieves high accuracy. The 95% CI for each modality further supports the robustness of the proposed model. Despite inherent differences between the METABRIC and TCGA datasets, the BiLSTM+CNN model achieves high accuracy across all data modalities. The results of our algorithm are compared with other state-of-the-art algorithms in Table 8 as follows:

**Table 8.** Comparison of classification performance of proposed Improved Deep Learning Algorithm (BiLSTM+CNN) with previous works on TCGA data.

Algorithm	Acc	Pre	Sn	Mcc
BiLSTM+CNN stacked RF	0.98	0.87	0.93	0.80
SiGaAtCNN Stacked RF [8]	0.91	0.84	0.80	0.77
Stacked RF [8]	0.92	0.83	0.80	0.76

## Discussion

The results confirm that BiLSTM+CNN, when combined with RF-based classification, offers significant improvements over existing algorithms. The algorithm achieved 98% accuracy, 1.0 sensitivity, 0.95 precision, and 0.81 MCC on METABRIC data, and 98% accuracy, 0.87 precision, 0.93 sensitivity, and 0.80 MCC on TCGA data.

The combination of CNN and BiLSTM allows the model to effectively handle both time-series and spatial data, enhancing predictive performance. However, challenges remain with gene expression data, which require further research and hyperparameter tuning. Nonetheless, the strong performance across multiple datasets supports the potential of this model for personalized treatment and clinical decision-making.

In terms of classification, the BiLSTM+CNN model outputs probabilities ranging between 0 and 1 for each class. To convert these probabilities into binary labels (0/1), a thresholding technique was employed. We used the validation set to determine the optimal threshold, selecting the value that maximized the AUC-ROC score. This approach ensures the best balance between sensitivity and specificity, especially when dealing with imbalanced class distributions. The same threshold was applied to the test set to compute the final performance metrics reported in this study. This threshold optimization ensures that the reported metrics—accuracy, sensitivity, specificity, and precision accurately reflect the model’s true performance under realistic conditions.

### Comparison with relevant literature

Our study builds upon existing research that utilizes multi-omics data for survival prediction. Curtis et al. ^13^ identified prognostic biomarkers using a multidimensional competition-based framework with the METABRIC dataset, while our study advances this work by integrating BiLSTM and CNN architectures for capturing temporal and spatial patterns across data modalities. Unlike the framework by Curtis et al., ^13^ which focused primarily on identifying subgroups, our model emphasizes multi-omics data integration for improved survival predictions and interpretability.

Additionally, Yousefi et al. ^7^ and Mobadersany et al. ^18^ employed convolutional networks for cancer survival outcome predictions, but their models primarily focused on histological data. In contrast, our model integrates clinical, CNA, and gene expression data, providing a more comprehensive and interpretable prediction framework. This integration allows the model to extract complex patterns that go beyond histological data alone. The study by Jadoon et al. ^26^ proposed a heterogeneous multiple kernel learning approach for breast cancer prognosis, addressing the challenge of multimodal data. While their approach is robust, our deep learning-based solution offers enhanced predictive performance through the combined use of BiLSTM and CNN architectures, which capture both sequential and spatial information across data types. Similarly, Phan et al. ^6^ demonstrated the use of machine learning models for decoding breast cancer with multi-omics data but faced challenges related to model interpretability and high dimensionality. Our approach, with MRMR feature selection, addresses these challenges by reducing dimensionality while retaining the most informative features.

In summary, our model offers a novel combination of deep learning models and feature selection techniques to provide actionable clinical insights. The use of decision-level integration ensures robust predictions across datasets, with significant improvements observed on both METABRIC and TCGA datasets. These comparisons highlight how our work advances the field by building on previous methodologies while addressing key limitations, such as the interpretability and scalability of predictive models.

## Conclusion

Over the past two decades, significant progress has been made in the treatment of primary breast cancer, with advancements in early detection, prognosis, and treatment leading to a notable decrease in mortality rates. However, breast cancer continues to pose challenges, particularly in terms of early detection and precise survival prediction. The heterogeneity in clinical outcomes and the complexity associated with genetic variations present challenges for oncologists in devising optimal treatment plans. Therefore, developing intelligent systems to enhance breast cancer diagnosis and treatment remains essential.

This research introduced an improved deep learning algorithm (BiLSTM+CNN) aimed at benefiting both individuals with breast cancer and healthcare practitioners. The proposed algorithm utilizes a stacked ensemble framework, combining BiLSTM and CNN for feature extraction and a boosted Random Forest (RF) for survival prediction. The study leverages multi-omics data, including clinical data, copy number alteration data, and gene expression data. These extracted features serve as input to the boosted RF classifier, resulting in superior survival prediction.

Our experimental results demonstrate that the proposed deep learning model (BiLSTM+CNN) outperformed existing models, achieving an accuracy of 98%. Furthermore, the versatility of the model suggests its applicability to other aggressive cancers such as cervical cancer, oral cancer, and lung cancer. By integrating multiple data modalities, the proposed approach enhances the robustness and reliability of predictions.

Future work could explore the inclusion of additional omics data, such as pathway data, gene methylation profiles, and miRNA expression. Expanding the model’s capability to handle various cancer types would also contribute to advancing personalized treatment approaches and clinical decision-making.

## Ethical considerations

Not applicable.

## Software availability

• Source code available from: https://github.com/NNasarudin/CNN-BiLSTM-for-Breast-Cancer-Survival-Prediction-Based-on-Multi-Omics-Data.git
• Archived software available from: https://doi.org/10.5281/zenodo.15964646
• License: GNU Lesser General Public License v3.0.

## Data Availability

Data used in this research is available in the cBioPortal for Cancer Genomics and Genomic Data Commons (GDC) Data Portal database.
• cBioPortal: Breast Cancer (METABRIC, Nature 2012 & Nat Commun 2016). https://www.cbioportal.org/study/summary?id=brca_metabric
• GDC Data Portal: Breast Invasive Carcinoma (TCGA-BRCA): Accession number: phs000178. https://portal.gdc.cancer.gov/projects/TCGA-BRCA

## References

[1] Bray F Ferlay J Soerjomataram I : Global cancer statistics 2018: GLOBOCAN estimates of incidence and mortality worldwide for 36 cancers in 185 countries. *CA Cancer J. Clin.* 2018;68(6):394–424. 10.3322/caac.21492 30207593

[2] Huang J : Global incidence and mortality of breast cancer: a trend analysis. *Aging.* Feb. 2021;13(4):5748–5803. 10.18632/aging.202502 33592581 PMC7950292

[3] Sopik V : International variation in breast cancer incidence and mortality in young women. *Breast Cancer Res. Treat.* Apr. 2021;186(2):497–507. 10.1007/s10549-020-06003-8 33145697

[4] Paulin F Santhakumaran DA : Extracting Rules from Feed Forward Neural Networks for Diagnosing Breast Cancer. *Artif. Intell. Syst. Mach. Learn.* 2009;1(4):Art. no. 4.

[5] Zhao L : DeepOmix: A scalable and interpretable multi-omics deep learning framework and application in cancer survival analysis. *Comput. Struct. Biotechnol. J.* Jan. 2021;19:2719–2725. 10.1016/j.csbj.2021.04.067 34093987 PMC8131983

[6] Phan JH Hoffman R Kothari S : Integration of multi-modal biomedical data to predict cancer grade and patient survival. *2016 IEEE-EMBS International Conference on Biomedical and Health Informatics (BHI).* Feb. 2016; pp.577–580. 10.1109/BHI.2016.7455963 PMC4969000 27493999

[7] Zhao M Tang Y Kim H : Machine Learning With K-Means Dimensional Reduction for Predicting Survival Outcomes in Patients With Breast Cancer. *Cancer Inform.* Jan. 2018;17:1176935118810215. 10.1177/1176935118810215 30455569 PMC6238199

[8] Goli S Mahjub H Faradmal J : Survival Prediction and Feature Selection in Patients with Breast Cancer Using Support Vector Regression. *Comput. Math. Methods Med.* 2016;2016(1):1–12. 10.1155/2016/2157984 27882074 PMC5108874

[9] Gevaert O Smet FD Timmerman D : Predicting the prognosis of breast cancer by integrating clinical and microarray data with Bayesian networks. *Bioinformatics.* Jul. 2006;22(14):e184–e190. 10.1093/bioinformatics/btl230 16873470

[10] Sun D Li A Tang B : Integrating genomic data and pathological images to effectively predict breast cancer clinical outcome. *Comput. Methods Prog. Biomed.* Jul. 2018;161:45–53. 10.1016/j.cmpb.2018.04.008 29852967

[11] Ma T Zhang A : Multi-view Factorization AutoEncoder with Network Constraints for Multi-omic Integrative Analysis. *2018 IEEE International Conference on Bioinformatics and Biomedicine (BIBM).* Dec. 2018; pp.702–707. 10.1109/BIBM.2018.8621379

[12] Martin LR Williams SL Haskard KB : The challenge of patient adherence. *Ther. Clin. Risk Manag.* Sep. 2005;1(3):189–199. 10.2147/tcrm.s12160382 18360559 PMC1661624

[13] Curtis C : The genomic and transcriptomic architecture of 2,000 breast tumours reveals novel subgroups. *Nature.* Jun. 2012;486(7403):346–352. 10.1038/nature10983 22522925 PMC3440846

[14] Arya N Saha S : Multi-modal advanced deep learning architectures for breast cancer survival prediction. *Knowl.-Based Syst.* Jun. 2021;221:106965. 10.1016/j.knosys.2021.106965

[15] Tomczak K Czerwińska P Wiznerowicz M : Review The Cancer Genome Atlas (TCGA): an immeasurable source of knowledge. *Contemp. Oncol. Onkol.* 2015;1A(1):68–77. 10.5114/wo.2014.47136 25691825 PMC4322527

[16] Moreno-Barea FJ Jerez JM Franco L : Gan-based data augmentation for prediction improvement using gene expression data in cancer. *International Conference on Computational Science.* Vol.13352. Cham; Springer International Publishing;2022 Jun; pp.28–42. 10.1007/978-3-031-08757-8_3

[17] Nouri N : Single-cell RNA-seq data augmentation using generative Fourier transformer. *Commun. Biol.* 2025 Jan 22;8(1):113. 10.1038/s42003-025-07552-8 39843603 PMC11754799

[18] Cheerla A Gevaert O : Deep learning with multimodal representation for pancancer prognosis prediction. *Bioinformatics.* Jul. 2019;35(14):i446–i454. 10.1093/bioinformatics/btz342 31510656 PMC6612862

[19] Vale-Silva LA Rohr K : Long-term cancer survival prediction using multimodal deep learning. *Sci. Rep.* Jun. 2021;11(1):13505. 10.1038/s41598-021-92799-4 34188098 PMC8242026

[20] Al-Helali B Chen Q Xue B : A new imputation method based on genetic programming and weighted KNN for symbolic regression with incomplete data. *Soft. Comput.* Apr. 2021;25(8):5993–6012. 10.1007/s00500-021-05590-y

[21] Tan J Hammond JH Hogan DA : Adage analysis of publicly available gene expression data collections illuminates pseudomonas aeruginosa-host interactions. *BioRxiv.* 2015 Nov 5:030650. 10.1128/mSystems.00025-15 PMC5069748 27822512

[22] Lecun Y Bottou L Bengio Y : Gradient-based learning applied to document recognition. *Proc. IEEE.* Nov. 1998;86(11):2278–2324. 10.1109/5.726791

[23] Hochreiter S Schmidhuber J : Long Short-Term Memory. *Neural Comput.* Nov. 1997;9(8):1735–1780. 10.1162/neco.1997.9.8.1735 9377276

[24] Ganaie MA Hu M Malik AK : Ensemble deep learning: A review. *Eng. Appl. Artif. Intell.* Oct. 2022;115:105151. 10.1016/j.engappai.2022.105151

[25] Kingma DP Ba J : Adam: A Method for Stochastic Optimization. Jan. 30, 2017. arXiv: arXiv:1412.6980. 10.48550/arXiv.1412.6980

[26] Jadoon EK Khan FG Shah S : Deep Learning-Based Multi-Modal Ensemble Classification Approach for Human Breast Cancer Prognosis. *IEEE Access.* 2023;11:85760–85769. 10.1109/ACCESS.2023.3304242

