# Supplementary material for: An Improved Deep Learning Algorithm for Breast Cancer Survival Prediction Based on Multi-Omics Data
Source: F1000Res. 2026 Apr 10;14:765. Originally published 2025 Aug 4. [Version 3] doi: 10.12688/f1000research.166682.3 (PMC13110385; doi:10.12688/f1000research.166682.3)
Supplement: Peer review history

# Reviewer report: Reviewer response for version 3

**DOI:** 10.5256/f1000research.197690.r474661

**Published:** 2026-04-27

**Authors:** Suhaila Zainudin

**Affiliations:** 1 Universiti Kebangsaan Malaysia, Bangi, Selangor, Malaysia

I have read the revised edition and have no further comments.

Is the work clearly and accurately presented and does it cite the current literature?

Partly

If applicable, is the statistical analysis and its interpretation appropriate?

Yes

Are all the source data underlying the results available to ensure full reproducibility?

Yes

Is the study design appropriate and is the work technically sound?

Yes

Are the conclusions drawn adequately supported by the results?

Yes

Are sufficient details of methods and analysis provided to allow replication by others?

Partly

Reviewer Expertise:

Data Analytics

I confirm that I have read this submission and believe that I have an appropriate level of expertise to confirm that it is of an acceptable scientific standard.

# Reviewer report: Reviewer response for version 3

**DOI:** 10.5256/f1000research.197690.r476836

**Published:** 2026-04-25

**Authors:** Toni Arifin

**Affiliations:** 1 Universitas Adhirajasa Reswara Sanjaya, Bandung, Indonesia

This technically robust paper demonstrates exceptional transparency following its third revision. By providing GitHub source code and documenting critical technical parameters—including random seeds, hardware specifications, and specific software versions—the authors ensure full reproducibility. This work offers a significant, verifiable contribution to the bioinformatics research community, effectively addressing previous concerns regarding methodological clarity and experimental replication standards.

Is the work clearly and accurately presented and does it cite the current literature?

Yes

If applicable, is the statistical analysis and its interpretation appropriate?

Yes

Are all the source data underlying the results available to ensure full reproducibility?

Yes

Is the study design appropriate and is the work technically sound?

Yes

Are the conclusions drawn adequately supported by the results?

Yes

Are sufficient details of methods and analysis provided to allow replication by others?

Yes

Reviewer Expertise:

Machine Learning, Image Processing, Deep Learning and Artificial Intelligence.

I confirm that I have read this submission and believe that I have an appropriate level of expertise to confirm that it is of an acceptable scientific standard.

# Reviewer report: Reviewer response for version 2

**DOI:** 10.5256/f1000research.187135.r445784

**Published:** 2026-02-17

**Authors:** Suhaila Zainudin

**Affiliations:** 1 Universiti Kebangsaan Malaysia, Bangi, Selangor, Malaysia

This article presents a hybrid deep learning framework (BiLSTM+CNN with stacked Random Forest) for predicting breast cancer survival using multi-omics data from the METABRIC and TCGA datasets. The authors integrate clinical, copy number alteration, and gene expression features with MRMR feature selection and evaluate performance using cross-validation and ROC-based metrics. The study reports high predictive accuracy and demonstrates external validation across datasets, suggesting robustness and potential clinical relevance.

 The manuscript is clearly structured and generally well written. The background appropriately situates the work within recent literature on multi-omics survival modeling. However, claims regarding interpretability and clinical applicability would benefit from more explicit demonstrations (e.g., feature importance analysis linked to biological pathways). This is a *recommended improvement*, not a requirement for scientific soundness.

 The use of two independent public datasets, cross-validation, and standard evaluation metrics constitutes an appropriate and technically sound design. External validation strengthens the conclusions. An ablation study isolating the contribution of each architectural component would further support claims of model superiority but is optional.

 While the authors provide architecture details and public code, several aspects limit full replication. To ensure scientific soundness, the authors **must**: (1) specify exact preprocessing pipelines and parameter settings for MRMR and data augmentation; (2) document all random seeds and data splits; and (3) describe the computational environment (hardware/software versions). Providing a step-by-step reproducibility protocol or configuration file would resolve these issues.

 The evaluation metrics and cross-validation strategy are appropriate. Including formal statistical comparisons between models or calibration analyses would strengthen rigor but is not essential.

 Public datasets and open code support reproducibility. The conclusions are broadly supported by the results, though statements about clinical impact should be moderated or accompanied by discussion of deployment limitations.

 Overall, the article is scientifically promising. Addressing the required reproducibility clarifications will make it methodologically robust.

Is the work clearly and accurately presented and does it cite the current literature?

Partly

If applicable, is the statistical analysis and its interpretation appropriate?

Yes

Are all the source data underlying the results available to ensure full reproducibility?

Yes

Is the study design appropriate and is the work technically sound?

Yes

Are the conclusions drawn adequately supported by the results?

Yes

Are sufficient details of methods and analysis provided to allow replication by others?

Partly

Reviewer Expertise:

Data Analytics

I confirm that I have read this submission and believe that I have an appropriate level of expertise to confirm that it is of an acceptable scientific standard, however I have significant reservations, as outlined above.

## References

[1] : Metaheuristic approach for an enhanced mRMR filter method for classification using drug response microarray data. *Expert Systems with Applications* .2017;90: 10.1016/j.eswa.2017.08.026 224-231 10.1016/j.eswa.2017.08.026

# Response

**Published:** 2026-03-12

**Authors:** Mohd Saberi Mohamad

**Affiliations:** Genetics and Genomics, United Arab Emirates University, Al Ain, Abu Dhabi, United Arab Emirates

Comment 1:

 However, claims regarding interpretability and clinical applicability would benefit from more explicit demonstrations (e.g., feature importance analysis linked to biological pathways). This is a *recommended improvement*, not a requirement for scientific soundness.

 Amendment 1:

 This part has been improved and revised to clarify the scope and limitations of the study. We explicitly acknowledged that biological interpretation of selected features and their association with breast cancer pathways was not explored, and we suggested future directions such as pathway enrichment or gene importance analyses to enhance interpretability. Additionally, we have highlighted key considerations and limitations regarding clinical applicability, including dataset constraints, preprocessing dependencies, model calibration, and the need for prospective validation, regulatory approval, and clinician evaluation.

 Under discussion section (paragraph 4 and 5)

 Comment 2:

 While the authors provide architecture details and public code, several aspects limit full replication. To ensure scientific soundness, the authors must:

 (1) specify exact preprocessing pipelines and parameter settings for MRMR and data augmentation;

 Amendment 2:

 This part has been improved and revised to ensure full reproducibility. We added a detailed flowchart of the preprocessing pipeline and explicitly described all preprocessing steps and parameter settings.

 Under preprocessing data section. Added figure 1 as the flowchart of the preprocessing.

 Explanation at paragraph 3 and 4.

 Comment 3:

 (2) document all random seeds and data splits;

 Amendment 3:

 All random seeds and data splits have been included.

 Under data augmentation section (last paragraph)

 Comment 4:

 (3) describe the computational environment (hardware/software versions).

 Amendment 4:

 Computational environment has been included.

 Under computational environment section.

 Comment 5:

 Public datasets and open code support reproducibility. The conclusions are broadly supported by the results, though statements about clinical impact should be moderated or accompanied by discussion of deployment limitations.

 Amendment 5:

 The explanation has been included, as detailed in point number 1

# Reviewer report: Reviewer response for version 2

**DOI:** 10.5256/f1000research.187135.r410019

**Published:** 2025-09-05

**Authors:** Weng Howe Chan

**Affiliations:** 1 Universiti Teknologi Malaysia, Johor Bahru, Malaysia

Authors have addressed all the comments, especially regarding data augmentations and the MRMR feature selection process. No further comments.

Is the work clearly and accurately presented and does it cite the current literature?

Yes

If applicable, is the statistical analysis and its interpretation appropriate?

I cannot comment. A qualified statistician is required.

Are all the source data underlying the results available to ensure full reproducibility?

Yes

Is the study design appropriate and is the work technically sound?

Yes

Are the conclusions drawn adequately supported by the results?

Yes

Are sufficient details of methods and analysis provided to allow replication by others?

Partly

Reviewer Expertise:

bioinformatics, computational biology, artificial intelligence

I confirm that I have read this submission and believe that I have an appropriate level of expertise to confirm that it is of an acceptable scientific standard.

# Reviewer report: Reviewer response for version 1

**DOI:** 10.5256/f1000research.183707.r402954

**Published:** 2025-08-12

**Authors:** Weng Howe Chan

**Affiliations:** 1 Universiti Teknologi Malaysia, Johor Bahru, Malaysia

- in section A, it is mentioned that there are 25 clinical features of METABRIC after remove the two with missing values and redundancy. Does all the 25 remaining features are examined with the t-test? but only part of them are showing in Table 2? Should clarify and justify this.

 - regarding data augmentation, should include justification how appropriate DA techniques are relevant in this case with gene expression, as this is not image. so how random rotation and noise injection would make sense here? the included reference 15, 16 seems doesn't reflect the existing works on this. Though in ref 15's future work did mention about data augmentation, but the support of use of DA here is very minimal. Should justify properly.

 - in this study, MRMR is used as feature selection, should explain more how the MRMR especially the parameter tuning is done, at least about the key settings of MRMR after the hyperparameter tuning and how these parameters would affect the outcome. This should be clarify as the rest of the learning is actually from the output features of this feature selection process.

Is the work clearly and accurately presented and does it cite the current literature?

Yes

If applicable, is the statistical analysis and its interpretation appropriate?

I cannot comment. A qualified statistician is required.

Are all the source data underlying the results available to ensure full reproducibility?

Yes

Is the study design appropriate and is the work technically sound?

Yes

Are the conclusions drawn adequately supported by the results?

Yes

Are sufficient details of methods and analysis provided to allow replication by others?

Partly

Reviewer Expertise:

bioinformatics, computational biology, artificial intelligence

I confirm that I have read this submission and believe that I have an appropriate level of expertise to confirm that it is of an acceptable scientific standard, however I have significant reservations, as outlined above.

# Response

**Published:** 2025-08-23

**Authors:** Mohd Saberi Mohamad

**Affiliations:** Genetics and Genomics, United Arab Emirates University, Al Ain, Abu Dhabi, United Arab Emirates

1.    in section A, it is mentioned that there are 25 clinical features of METABRIC after remove the two with missing values and redundancy. Does all the 25 remaining features are examined with the t-test? but only part of them are showing in Table 2? Should clarify and justify this.

This point has been clarified and justified on page 5.

 2.    regarding data augmentation, should include justification how appropriate DA techniques are relevant in this case with gene expression, as this is not image. so how random rotation and noise injection would make sense here? the included reference 15, 16 seems doesn't reflect the existing works on this. Though in ref 15's future work did mention about data augmentation, but the support of use of DA here is very minimal. Should justify properly.    

 This point has been clarified on page 7 and 8.

The justification for applying data augmentation techniques to gene expression data, including the relevance of random rotation and noise injection in this context, has been provided.

 3.    in this study, MRMR is used as feature selection, should explain more how the MRMR especially the parameter tuning is done, at least about the key settings of MRMR after the hyperparameter tuning and how these parameters would affect the outcome. This should be clarify as the rest of the learning is actually from the output features of this feature selection process.  

Further explanation has been added regarding the use of MRMR on page 8 and 9.

# Response

**Published:** 2025-08-31

**Authors:** Mohd Saberi Mohamad

**Affiliations:** Genetics and Genomics, United Arab Emirates University, Al Ain, Abu Dhabi, United Arab Emirates

Thank you for taking the time to read our article, *“An Improved Deep Learning Algorithm for Breast Cancer Survival Prediction Based on Multi-Omics Data.”* In this work, we aimed to develop an interpretable and effective deep learning framework that integrates BiLSTM, CNN, and MRMR feature selection for breast cancer survival prediction.

 We hope that the findings, particularly the accuracy and robust performance across the METABRIC and TCGA datasets, will contribute to ongoing efforts in improving personalised treatment planning and clinical decision-making. We welcome constructive feedback, discussions, and collaborations that can further enhance the clinical translation of this approach.
